# Moss Pathogenesis-Related-10 Protein Enhances Resistance to *Pythium irregulare* in *Physcomitrella patens* and *Arabidopsis thaliana*

**DOI:** 10.3389/fpls.2016.00580

**Published:** 2016-04-29

**Authors:** Alexandra Castro, Sabina Vidal, Inés Ponce de León

**Affiliations:** ^1^Departamento de Biología Molecular, Instituto de Investigaciones Biológicas Clemente EstableMontevideo, Uruguay; ^2^Laboratorio de Biología Molecular Vegetal, Facultad de Ciencias, Universidad de la RepúblicaMontevideo, Uruguay

**Keywords:** *Physcomitrella patens*, pathogenesis-related protein 10, *Pythium irregulare*, Arabidopsis thaliana, defense, cell wall

## Abstract

Plants respond to pathogen infection by activating signaling pathways leading to the accumulation of proteins with diverse roles in defense. Here, we addressed the functional role of *PpPR-10*, a pathogenesis-related (PR)-10 gene, of the moss *Physcomitrella patens*, in response to biotic stress. *PpPR-10* belongs to a multigene family and encodes a protein twice the usual size of PR-10 proteins due to the presence of two Bet v1 domains. Moss *PR-10* genes are differentially regulated during development and inoculation with the fungal pathogen *Botrytis cinerea*. Specifically, *PpPR-10* transcript levels increase significantly by treatments with elicitors of *Pectobacterium carotovorum* subsp. *carotovorum*, spores of *B. cinerea*, and the defense hormone salicylic acid. To characterize the role of PpPR-10 in plant defense against pathogens, we conducted overexpression analysis in *P. patens* and in *Arabidopsis thaliana*. We demonstrate that constitutive expression of *PpPR-10* in moss tissues increased resistance against the oomycete *Pythium irregulare*. PpPR-10 overexpressing moss plants developed less symptoms and decreased mycelium growth than wild type plants. In addition, PpPR-10 overexpressing plants constitutively produced cell wall depositions in protonemal tissue. Ectopic expression of *PpPR-10* in *Arabidopsis* resulted in increased resistance against *P. irregulare* as well, evidenced by smaller lesions and less cellular damage compared to wild type plants. These results indicate that PpPR-10 is functionally active in the defense against the pathogen *P. irregulare*, in both *P. patens* and *Arabidopsis*, two evolutionary distant plants. Thus, *P. patens* can serve as an interesting source of genes to improve resistance against pathogen infection in flowering plants.

## Introduction

Flowering plants have developed various defense strategies to cope with invading pathogens. As part of the defense responses, both environmental and pathogen-derived signals can be perceived and activate the expression of defense genes via different signaling pathways. Some of the genes involved in defense encode PR proteins (van Loon et al., 2006). Genes encoding these proteins are generally induced by different types of pathogens, such as bacteria, fungi, oomycetes, and viruses (van Loon et al., 1994). PR proteins accumulate at the infection site in response to pathogen infection and contribute to systemic acquired resistance (SAR) (Ryals et al., 1996). These proteins are spread throughout the plant kingdom and divided into 17 classes (PR-1-17) on the basis of their amino acid sequence identity, biological activity or physicochemical properties (van Loon et al., 2006). Several members of the PR protein family have enzymatic activities, including β-1,3-glucanase (PR-2), chitinase (PR-3, -4, -8, and -11), peroxidase (PR-9) or endoproteinase (PR-7), and have shown to exhibit either antibacterial or antifungal activity (Edreva, 2005; van Loon et al., 2006).

Mosses are primitive land plants that have diverged from the ancestors of the flowering plants early in land plant evolution. As part of the transition of plants from water to land, mosses have developed adaptations strategies to cope with radiation, drought, and extreme temperatures (Rensing et al., 2008), as well as airborne pathogen attack and insect/animal predation. In the last decade, the moss *P. patens* has become an attractive plant model to study defense mechanisms activated in response to biotic and abiotic stress (Frank et al., 2005; Rensing et al., 2008; Ponce de León and Montesano, 2013). Since mosses are an evolutionary link between green algae and tracheophytes, studies in *P. patens* can provide new insights into the evolution of plant defense mechanisms against different types of stress. Several proteins involved in desiccation, salt and osmotic stress tolerance have been identified in this moss, including dehydrins, a TSPO1 protein involved in mitochondrial tetrapyrrole transport, small heat shock proteins, a Ca2+-ATPase, an actinoporin and an ATP binding cassette transporter (Saavedra et al., 2006; Frank et al., 2007; Qudeimat et al., 2008; Hoang et al., 2009; Ruibal et al., 2012, 2013; Buda et al., 2013). Some of these studies have revealed novel mechanisms involved in stress tolerance, showing the potential use of this moss in deciphering the function of defense proteins. Up to date only few defense-related proteins have been identified in *P. patens*. Functional studies have shown that a peroxidase (Prx34) and TSPO1 are important for plant resistance against saprophytic and pathogenic fungi (Lehtonen et al., 2009, 2012). The oxylipin forming alpha-Dioxygenase participates in the defense against the necrotrophic fungus *B. cinerea*, and protects plant tissues from damage caused by elicitors of the necrotrophic bacteria *P.c. carotovorum* (Machado et al., 2015). In addition to Prx34, which is induced by fungal pathogens, a gene encoding a PR-1 is induced in this moss in response to elicitors of *P.c. carotovorum, B. cinerea*, and two necrotrophic *Pythium* species (oomycetes) (Ponce de León et al., 2007; Oliver et al., 2009). Other genes encoding proteins involved in defense such as lipoxygenases, allene oxide synthase, phenylalanine ammonia-lyase, chalcone synthase are also induced in *P. patens* in response to pathogen attack (Ponce de León et al., 2007, 2012; Oliver et al., 2009; Reboledo et al., 2015).

In order to identify more genes involved in defense responses against elicitors of *P.c. carotovorum* in *P. patens*, suppression subtractive hybridization was employed to construct a cDNA library enriched in sequences representing genes induced by *P.c. carotovorum* elicitors. One of the sequence present in the subtractive library corresponded to a gene encoding a PR-10 protein (Phypa_105033), designated here as PpPR-10. This class of PR proteins, also known as Bet v1 proteins, are generally small (17 kDa), acidic and cytosolic proteins (Grote, 1999; Ekramoddoullah, 2004; Chadha and Das, 2006). A hydrophobic binding domain, which binds diverse ligands, has been identified in several PR-10 proteins (Mogensen et al., 2002; Fernandes et al., 2013). PR-10 genes are differentially regulated in flowering plants during development and in response to pathogen infection, drought, and salinity (Dubods and Plomion, 2001; Liu and Ekramoddoullah, 2006; Jain et al., 2006; Jellouli et al., 2010). Phytohormones such as cytokinins, ABA, jasmonic acid (JA), SA, auxin and ethylene induce PR-10 genes in flowering plants (Fernandes et al., 2013). The precise function of PR-10 proteins is poorly understood. In *P. patens*, PpPR-10 was previously identified as a protein of 290 amino acids, which binds cytokinin (UBP34; urea-type cytokinin-binding protein of 34 kDa) (Gonneau et al., 2001). PpPR-10 has high similarity with major pollen allergen BetV1 proteins (Breiteneder et al., 1989), and contains a conserved glycine-rich GXGXXG motif present in all PR-10 proteins (Saraste et al., 1990; Gonneau et al., 2001). In addition, PpPR-10 has two tandemly arranged BetV1 motifs that share 45% sequence identity (Gonneau et al., 2001). PpPR-10 knockout mutants do not have a visible morphological phenotype and respond similarly to osmotic and salt stress compared to wild type plants, probably due to functional redundancy of other PpPR-10 proteins (Brun et al., 2004). In this study, we addressed the functional role of PpPR-10 in moss defense against pathogen infection by using an overexpression approach. The results showed that overexpression of this gene in *P. patens* increased resistance against *P. irregulare*. Moreover, heterologous expression of PpPR-10 in *Arabidopsis* increased resistance of transgenic lines against this oomycete.

## Materials and Methods

### Plant Material and Growth Conditions

*Physcomitrella patens* Gransden wild type isolate was grown and maintained on cellophane overlaid agar BCDAT medium (1.6 g L^-1^ Hoagland’s, 1 mM MgSO_4_,1.8 mM KH_2_PO_4_ pH 6.5, 10 mM KNO_3_,45 μM FeSO_4_, 1 mM CaCl_2_, 5 mM ammonium tartrate and 10 g L^-1^ agar) as described by Ashton and Cove (1977). Moss colonies and protonemal cultures were cultivated in a growth room as described previously (Oliver et al., 2009). Plants were grown at 22°C under a photoperiod of 16 h light and a photon flux of 60 μmol m^-2^ sec^-1^, and 3-week-old colonies were used for all the experiments. *Arabidopsis thaliana* (Col-0) and transgenic PpPR-10 lines (T3 and T4 plants) were grown on soil in a growth room at 24°C under a photoperiod of 16 h light and a photon flux of 100 μmol m^-2^ s^-1^. All experiments were repeated at least three times.

### Pathogen Inoculation, Culture Filtrates, and Hormones Treatments

*Pectobacterium carotovorum* subsp. *carotovorum* strain SCC1 (Rantakari et al., 2001) and *Pectobacterium wasabiae* strain SCC3193 (Nykyri et al., 2012) were propagated on Luria-Bertani (LB) medium at 28°C. Cell-free culture filtrates (CF) were prepared according to Ponce de León et al. (2007). The CF containing the elicitors was applied by spraying the moss colonies. *B. cinerea* was cultivated on 24 g/L PDA (Difco) at 22°C and inoculated by spraying a 2 × 10^5^ spores/mL suspension in water. As control water-treated plants were used. *P. patens* colonies were inoculated with 0.5 cm agar plugs containing *P. irregulare* mycelium as described by Oliver et al. (2009). Detached *Arabidopsis* leaves were placed in Petri dishes containing a wet filter paper and each leaf was inoculated with 0.5 cm diameter PDA plugs containing *P. irregulare*. As control PDA plugs were placed on top of each moss colony or *Arabidopsis* leaf. For hormones treatments, 3-weeks-old *P. patens* colonies were transferred to medium supplemented with the indicated final concentrations (SA, ABA, and NAA). MeJA was sprayed on top of the moss colonies.

### Sequence Alignment, Three-dimensional Structure Modeling, and Phylogenetic Analysis of Plant PR-10 Proteins

Protein sequence alignment was realized using the ClustalW program (Thompson et al., 1997). Full-length amino acid sequences from PR-10 proteins were retrieved from Phytozome. The three-dimensional models of the complete amino acid sequence of PpPR-10 were created with I-TASSER web server^1^ (Zhang, 2008; Yang et al., 2015). Sequences were aligned with ClustalW in a MEGA version 5.05 software for subsequent phylogenetic analysis (Tamura et al., 2011). Construction of phylogenetic tree was done using the Neighbor joining algorithm (Saitou and Nei, 1987). The percentage of replicate trees is shown on the branches and it is calculated in the bootstrap test (1000 replicates) for the associated taxa being clustered together.

### PpPR-10 Subcellular Localization

*PpPR-10* full length cDNA without the stop codon was amplified from *P. patens* moss colonies treated during 4 h with elicitors of *P.c. carotovorum* strain SCC1 using primers PpPR-10Lf (forward primer: 5′-CTTGGATCCATGGCCCACACTCTCTCCC-3′) and PpPR-10Lr (reverse primer: 5′-CGAGTGCGGCCGCGCGGCGGCAGCAGCCTC-3′) which contained restriction sites for *Bam*HI and *Not*I, respectively. The corresponding fragment was cloned into *Bam*HI and *Not*I sites of the pENT vector, and transferred via LR clonase (Gateway, Invitrogen) to vector pK7FWG2 containing the GFP coding sequence under the regulation of the 35S promoter (Karimi et al., 2002). The *PpPR-10* cDNA sequence was fused in frame to the 5′ end of GFP and the resulting construct was introduced in *Agrobacterium tumefaciens* strain pGV3101 by tri-parental mating (Wise et al., 2006). *Nicotiana tabacum* leaves were agroinoculated with the construct 35S:PR-10-GFP as described by Yang et al. (2000). Confocal imaging was performed using a confocal laser scanning microscope Leica TCS SP5 (Leica Microsystems Brasil, Sao Paulo, Brasil), with an excitation of 488 nm for GFP using software package LASAF v2.6.0 (Leica Microsystems, Mannheim, Germany).

### PpPR-10 Overexpression

For generation of overexpressing PpPR-10 plants, the cDNA was amplified from elicitor-treated moss tissues as described previously using primers PpPR-10f (forward primer: 5′-TCGCCGTGTGGGGATCCGTTGAG-3′) and PpPR-10r (reverse primer: 5′-TCCATACATGCGGCCGCGTCACCATG-3′) which contained restriction sites for *Bam*HI and *Not*I, respectively. The corresponding fragment was cloned into *Bam*HI and *Not*I sites of the pENT vector, and transferred via LR clonase (Gateway, Invitrogen) to a pTHUbi destination vector (kindly provided by Pierre-Francois Perroud, University of Washington, USA) (Perroud et al., 2011).

### Preparation of Protoplasts and Transformation of *P. patens*

Moss protoplasts were transformed with pUBI:PpPR-10 linearized with *Sw*aI and targeted to the genomic 108 locus (Schaefer and Zryd, 1997). Polyethylene glycol-mediated transformation of protoplasts was performed as described by Schaefer et al. (1991). Protoplasts (final concentration of 1 × 10^6^) were incubated with at least 15 μg of digested DNA and plated on BCDAT medium supplemented with 10 mM CaCl_2_ and 0.44 M mannitol. Protoplasts were thereafter transferred to BCDAT medium supplemented with 25 μg mL^-1^ of hygromycin and cultured for 7 days. Protoplasts were subsequently allowed to grow for 7 days on BCDAT medium without selection and finally returned to BCDAT medium containing 25 μg mL^-1^ of hygromycin. Tissues of plants showing growth after 3 weeks of selection were harvested for analyzing the incorporation of the transgene. Levels of *PpPR-10* transcript accumulation of the selected transformants were assayed by Northern blot analysis as described below.

### Flow Cytometry to Measure DNA Content

Two-week-old colonies were chopped with a razor blade in a Petri dish with 1 ml of nuclei extraction buffer (WPB) containing 0.2 M Tris-HCl pH 7.5, 4 mM MgCl_2_, 2 mM EDTANa_2_, 86 mM NaCl, 1% Triton X-100, 10 mM K_2_O_5_S_2_, and 1% PVP-10, and incubated for 15 min on ice. The resulting suspension was filtered through a 50 μm nylon mesh and incubated with 50 μl of Propidium Iodide (PI) (1 mg/mL, final concentration 50 μg/mL) and 50 μl of RNase A (1 mg/mL, final concentration 50 μg/mL) for 10 min at room temperature, to stain the DNA and to avoid double stranded RNA staining. Flow cytometry was performed according to Machado et al. (2015). Wild type samples of *P. patens* were used as an external control of DNA ploidy level.

### Northern Blot Analysis

Total RNA was isolated from *P. patens* plants using standard procedures based on phenol/chloroform extraction followed by LiCl precipitation. 10 μg of total RNA samples (each consisting of 48 colonies) were separated, transferred to nylon membranes (Hybond-N+, Amersham, GE Healthcare), and hybridized according to Ponce de León et al. (2007). *PpPR-10* cDNA was labeled with [α^32^P]-dCTP using the Rediprime II Random Prime labeling system (GE Healthcare, Buckinghamshire, UK). Ethidium bromide staining was used to ensure equal amounts of loading of RNA in each sample. The blots shown are representative examples of the result obtained in three independent experiments.

### Quantitative PCR

Total DNA was isolated from moss colonies inoculated with *P. irregulare* using the DNeasy kit (Qiagen, Hilden, Germany). Samples were frozen in liquid nitrogen at 2 or 24 h after inoculation. DNA was isolated from three independent experiments and each sample consisted of 16 *P. irregulare*-inoculated moss colonies. *P. patens* DNA corresponding to the single copy 3′-end of an elongation factor gene was amplified using the primers 5′-TTTGGGATTGAAATGTCGTG-3′ (EFf, forward) and 5′-TGAGCATGAGAAATTGGGTCT-3′ (EFr, reverse). *P. irregulare* DNA corresponding to the multiple copy ITS region was amplified using the primers 5′-ATCTGTGTTTTTGCATACTTGTGT-3′ (ITSf, forward) and 5′-TCCTCCGCTTATTGATATGC-3′ (ITSr, reverse). Quantitative PCR was performed using the QuantiMix Easy SYG kit (Biotools, Madrid, Spain) based on Sybr Green technology and the Rotor-Gene 6000 cycler (Corbett Life Science, Sydney, Australia). The annealing temperature was 55°C. The relative amount of *P. irregulare* DNA was normalized to plant DNA and expressed relative to the calibrator samples taken at 2 h. The ratio of *P. irregulare* to *P. patens* genomic DNA was calculated using the ΔΔCt method (Pfaﬄ, 2001).

### Colony Size Measurements of Wild Type and PpPR-10 Overexpressing Lines

Plant growth was monitored by measuring the colony diameters of 21 individual colonies grown during 3 weeks on BCDAT medium. The colony diameter was measured using GIMP 2.6 software^2^ (GNU Image Manipulation Program). To compare the significance of the differences between the diameters of the colonies a non-parametric Kruskal–Wallis multiple comparison test was performed using STATISTICA7 software. The measurements were repeated trice with similar results.

### Visualization of Plant Cell Wall-associated Defense Responses

Cell wall modifications were detected with solophenyl flavine 7GFE 500 and methyl blue for callose deposition according to Oliver et al. (2009). Bright field microscopy and fluorescence microscopy were performed with an Olympus BX61 microscope (Shinjuku-ku, Tokyo, Japan), and all images shown in this study were captured with the Cell F or MICROSUITE software package (Olympus, Tokyo, Japan).

### H_2_O_2_ and O_2_^-^ Detection

For H_2_O_2_ accumulation, staining of plant tissues was performed according to Thordal-Christensen et al. (1997). Moss colonies were immersed in freshly prepared 0.1% 3,3′-diaminobenzidine (DAB)-HCl (pH 3,8), vacuum infiltrated, and incubated in darkness at 22°C for 6 h. After incubation, moss colonies were bleached in an acetic acid:glycerol:ethanol (1:1:3) solution at 95°C for 10 min. For O_2_^-^ detection, staining was performed according to Trujillo et al. (2004). Moss colonies were placed in 0.1% nitroblue tetrazolium (NBT) in 10 mM potassium phosphate buffer (pH 7,8) containing 10 mM NaN_3_, vacuum infiltrated and incubated in darkness at 22°C for 2 h. Stained colonies were placed in a solution containing acetic acid:glycerol:ethanol (1:1:3) and boiled for 5 min. All tissues were stored at 95% ethanol until photographed.

### Cell Death Measurements

For cell death measurements, moss colonies were incubated with 0.05% Evans blue and after 1 h tissues were washed four times with deionized water to remove excess and unbound dye. Dye bound to dead cells was solubilized in 50% methanol with 1% SDS for 45 min at 50°C and the absorbance measured at 600 nm (Levine et al., 1994). Eight samples containing four colonies were analyzed per experiment, and each experiment was repeated at least three times and expressed as OD/mg dry weight.

### *Arabidopsis* Transformation and Molecular Characterization of the Transformed Lines

The PpPR-10-GFP construct was used to transform wild type *Arabidopsis* Col-0 by *Agrobacterium*-mediated flower dipping (Clough and Bent, 1998). Seeds were surface sterilized for 15 min in 7% of bleach with 0.05% Tween-20, washed five times with sterile water, plated in Petri dishes with half strength MS medium (2.4 g L^-1^ Murashige and Skoog, 5 g L^-1^ sucrose, 0.5 g L^-1^ monohydrate 2-ethanesulfonic acid and 10% agar) supplemented with 100 μg/mL kanacymin and incubated at 4°C for 3 days (stratification). Two-week-old plants were transferred to pots under described conditions until plants formed seeds. To select homozygous lines, T2 generation seeds were analyzed for germination on Km. Homozygous T3 and T4 plants were used for phenotypic analysis. Expression of the transgene and presence of the PpPR-10-GFP fusion protein was tested in 10 independent lines by semi-quantitative RT-PCR analysis and Western blot analysis. For semi-quantitative RT-PCR ubiquitin (gene AT3G52590) was used as an internal control. Total RNA was extracted from 14-day-old seedlings grown on half MS plates. For cDNA synthesis, 2 μg of total RNA was treated with RNasefree DNase I (Thermo Scientific) to remove genomic DNA contamination. cDNA was synthesized from total RNA using RevertAid Reverse transcriptase (Thermo Scientific) and oligo (dT) according to the manufacturer’s protocol. From the resulting 25 μL of cDNA, 2 μL were used as a template for PCR analysis using PpPR10 specific primers (PpPR-10f and PpPR-10r). Three independent PpPR-10 overexpressing *Arabidopsis* lines derived from the transformation were selected for Western blot analysis.

### Western Blot Analysis

For Western bloting, total protein extracts were prepared using the trichloroacetic acid (TCA)-acetone precipitation method essentially as described by Méchin et al. (2007). Briefly, approximately 200 mg of plant tissue were frozen in liquid nitrogen and grinded in a bead-beater using 2 mm zirconia beads. 1.8 mL of cold TCA-2-mercaptoethanol (2ME)-acetone solution [10% TCA (w/v), 0.07% 2ME (w/v) in cold acetone] was added and tubes were stored at -20°C for 1 h. After this period, samples were centrifuged for 10 min at 10,000 g at 4°C. The pellet was washed three times with rinsing solution [0.07% 2ME (w/v) in cold acetone] and stored for 1 h at -20°C every time. Finally, the pellet was dried in the laminar flow to fully eliminate the acetone and precipitated proteins were resuspended in solubilization solution (7 M urea, 2 M thiourea, 2% 3[3-cholaminopropyl diethylammonio]-1-propane sulfonate (CHAPS; w/v), 16 mM dithiothreitol (DTT) in doubledistilled (dd)H_2_O). Cellular debris were eliminated centrifuging for 15 min at 10,000 *g* at 25°C. Ten micrograms of soluble proteins were separated in 9% polyacrylamide gels and electroblotted onto nitrocellulose membranes (Hybond-ECL, Amersham, GE Healthcare). To ensure equal loading of protein samples, blots were stained with 0.5% Ponceau red. Membranes were blocked in Tris-buffered saline (TBS, 20 mM Tris-HCl, 150 mM NaCl pH 7.4) containing 5% skimmed milk powder and 0.2% Tween-20 for 1 h at room temperature. The commercial antibody against GFP (Sigma–Aldrich), was diluted 1/4000 in TBS, 0.1% Tween. Horseradish peroxidase-labeled goat anti-rabbit antibody (Sigma–Aldrich) diluted 1/10,000 in TBS-Tween 0.1% was used as secondary antibody. Protein reactions were visualized in autoradiography films using the ECL detection system.

### Ion Leakage

Membrane permeability of *Arabidopsis* leaves after *P. irregulare* inoculation was measured by electrolyte leakage. Leaves were rinsed three times with deionized water to remove surface-adhered electrolytes, then placed in groups of three in tubes containing 7.5 mL of deionized water and incubated at 25°C on an orbital shaker for 15 min at low speed (40 rpm). Samples were thereafter boiled for 30 min to disrupt the cellular membranes and then acclimated at room temperature. Conductivity was measured before (C1) and after cellular disruption (C2) using an Orion model 105 (Orion Research, Inc., USA) conductivity meter. Electrolyte leakage was calculated using the following equation: relative electrolyte leakage (%) = (C1/C2) × 100. Experiment was repeated twice with nine leaves per genotype.

## Results

### Alignment of Moss PR-10 Proteins and Phylogenetic Relationship with Other Plant PR-10 Proteins

**FIGURE 1:**
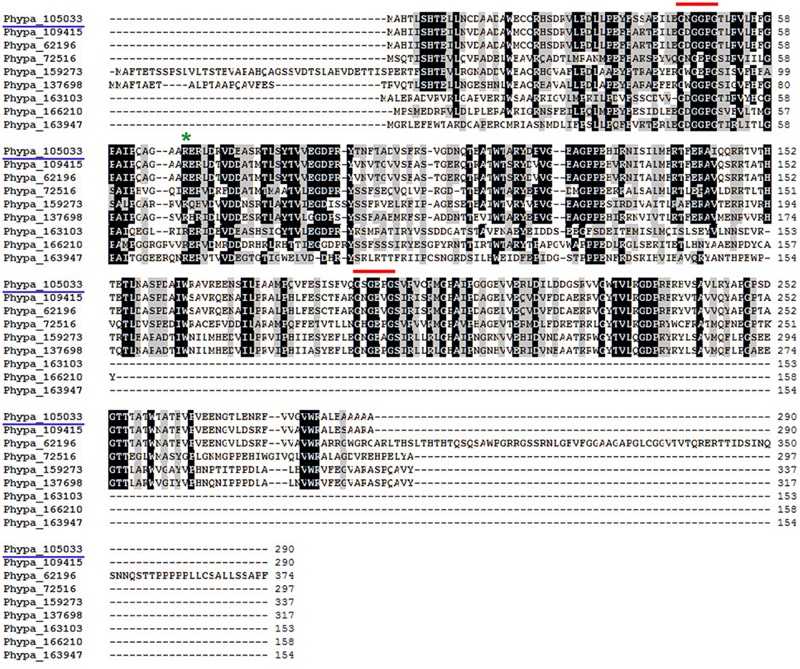
**Alignments of deduced amino acid sequences of PpPR-10 with other moss PR-10-like proteins.** Alignments were performed with ClustalW. PpPR-10 (Phypa_105033) used in this study is highlighted (blue underline). Red lines and the green asterisk mark the GXGXXG motif and the conserved lysine (K68), respectively.

A search in the *P. patens* public database Phytozome v10.3 revealed the existence of five additional genes encoding putative PR-10, including Phypa_109415, Phypa_62196, Phypa_72516, Phypa_159273, and Phypa_137698. The encoded proteins have amino acid sequence similarities ranging from 64,8 to 85,2% to PpPR-10. Two of these genes, Phypa_109415 and Phypa_62196, are identical to each other and are very closely located in chromosome 7, suggesting a gene duplication event. The high sequence similarity present in the different members of the PR-10 family of *P. patens* is consistent with the lack of phenotype observed in the PpPR-10 knockout mutant (Brun et al., 2004). PpPR-10 and PpPR-10-like proteins have two Bet v1 domains, each of them containing the conserved glycine-rich GXGXXG motif (**Figure 1**), also known as phosphate binding loop (Saraste et al., 1990). The deduced PpPR-10 and PpPR-10-like proteins ranged from 290 to 374 amino acids (**Figure 1**), which are twice the usual size described for other members of this class. The conserved lysine residue located 18 amino acid residues downstream the glycine-rich motif was only found in the first Bet v1 domain of PpPR-10 and PpPR-10-like proteins. In addition, we found three other genes encoding smaller proteins that contain a single Bet VI domain, including one GXGXXG motif (**Figure 1**). The deduced amino acid sequences of these genes, Phypa_166210, Phypa_163103, and Phypa_163947, exhibit 23,4, 25,9, and 25,9% similarities to PpPR-10, respectively. Only the protein encoded by Phypa_163947 has the conserved lysine residue (K68). PR-10 proteins have usually a fold consisting of three α helix motifs and seven antiparallel β strands arranged to form a large internal hydrophobic cavity involved in ligand binding (Fernandes et al., 2013). Modeling the three-dimensional structure of PpPR-10 using I-TASSER server (Zhang, 2008; Yang et al., 2015), suggests that the protein has two structurally distinct domains, each of them containing three α helix segments and seven β strands, which match the Bet v1 motifs (**Supplementary Figure S1**). The phylogenetic relationship between the PR-10 family from *P. patens* and other plant PR-10 was analyzed using ClustalW sequence alignment followed by the neighbor-joining algorithm employing the MEGA 5.05 program (**Supplementary Figure S2**). PpPR-10 and PpPR-10-like deduced proteins were compared to the most similar PR-10 proteins from vascular plants. The tree shows two clear clusters, one represented by *P. patens* PpPR-10 and PpPR-10-like proteins and two other PR-10 proteins from the lycophyte *Selaginella moellendorffii*, and a second cluster that groups the PR-10 proteins from flowering plants and one PR-10 from *S. moellendorffii*. The *P. patens* clade contains one cluster represented by the PpPR-10 proteins with two Bet v1 domains.

### PpPR-10 Is Localized in the Cytosol

**FIGURE 2:**
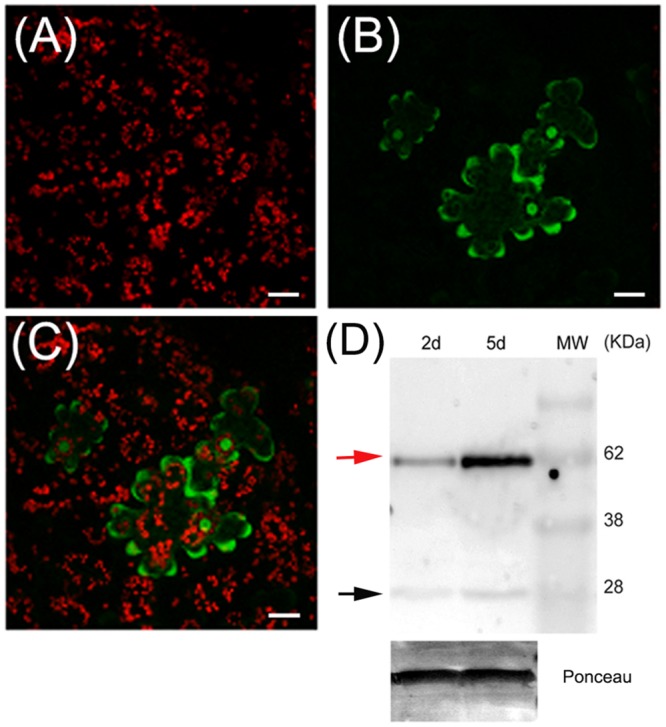
**Subcellular localization of PpPR-10 in agroinfiltrated tobacco leaves.** Confocal microscopy images of tobacco leaves agroinfiltrated with the 35S:PpPR-10-GFP construct. Leaves have been imaged 2 days after agroinfiltration by confocal microscopy. Red fluorescence belongs to chlorophyll in chloroplasts **(A)**, green fluorescence belongs to GFP **(B)**, and merged fluorescences are shown in **(C)**. **(D)** Immunoblot detection of PpPR-10-GFP fusion protein (red arrow) in agroinfiltrated tobacco leaves, showing that the signal is mostly derived from the fusion protein. Ten micrograms of total soluble proteins were separated in SDS-PAGE and Western blot analysis was performed using an antibody for GFP. The 27 kDa protein corresponds to cleaved GFP (black arrow). The *scale bars* represent 20 μm.

To determine the intracellular localization of PpPR-10, the coding region of the gene was fused in frame to the GFP and transiently expressed under the control of the constitutive CaMV 35S promoter (35S:PpPR-10-GFP) by agroinfiltrating tobacco leaves (**Figure 2**). Confocal microscopy showed that the fluorescent signal of PpPR-10-GFP fusion proteins was homogenously distributed in the cytosol of tobacco cells (**Figure 2**). Since GFP signal was also observed in the nucleus of transformed cells, we investigated whether this could be due to nuclear accumulation of free GFP produced by proteolytic cleavage of PpPR-10-GFP fusion proteins. Total protein extracts were isolated from tobacco agroinfiltrated leaf tissues overexpressing the PpPR-10-GFP and analyzed by Western blot using antibodies against GFP (**Figure 2D**). The results showed that although the majority of the signal corresponded to intact PpPR-10-GFP fusion protein (58 kDa), a band corresponding to the low molecular weight GFP (∼27 kDa) was also detected. This data strongly suggests that nuclear accumulation of GFP fluorescence is most likely due to the import of free GFP rather than the fusion protein and confirms a subcellular distribution of this protein similar to other members of the PR-10 protein family from flowering plants (Chadha and Das, 2006).

### Expression Profile of *PpPR-10* during Development and Pathogen Infection

**FIGURE 3:**
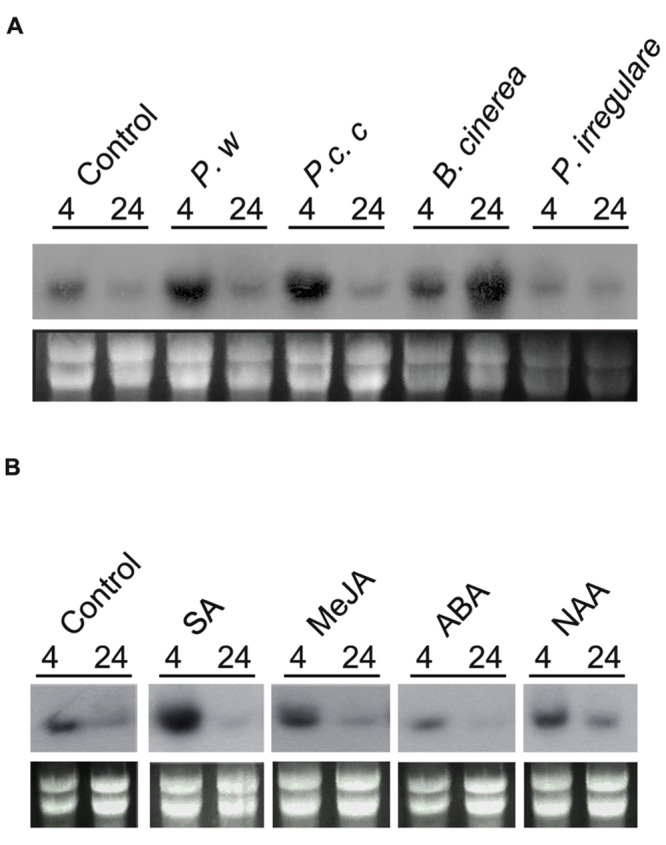
***PpPR-10* expression in response to pathogens and hormones. (A)** Expression of *PpPR-10* in response to elicitors of *P. wasabiae* (*P. w*) and *P.c. carotovorum* (*P.c.c*), spores of *B. cinerea* and mycelium of *P. irregulare* at different hours after treatments. As control colonies were treated with water. **(B)** Expression of *PpPR-10* in moss colonies treated with SA, MeJA, ABA, and auxin 1-naphthalene acetic acid (NAA). Ten micrograms of total RNA were separated on formaldehyde–agarose gels, transferred to a nylon membrane and hybridized to a ^32^P-labeled PpPR-10 cDNA probe. Ethidium bromide staining of rRNA was used to ensure equal loading of RNA samples. Experiments were repeated thrice with similar results.

To gain insight into the role of PpPR-10 during development and biotic stress, we monitored transcript levels in moss colonies exposed to different pathogens and after treatment with hormones involved in plant defenses. Control gametophytes have a basal expression of *PpPR-10* in Northern blot analysis (**Figure 3**). Analysis of digital expression profiles of different developmental stages of *P. patens* using microarray based expression data available at Genevestigator (Zimmermann et al., 2008)^3^, were consistent with the observed low level of constitutive expression of *PpPR-10* in gametophytes, and indicated a similar expression profile under control conditions for the other members of the *PpPR-10-like* gene family (**Supplementary Figure S3**). In addition, *PpPR-10* genes, except Phypa_159273, and Phypa_72516, appear to have a basal expression in the sporophyte and during spore germination (**Supplementary Figure S3**). To analyze *PpPR-10* mRNA accumulation in response to biotic stress, moss plants were treated with elicitors of two phytopathogenic bacteria of the genus *Pectobacterium*, and inoculated with the fungus *B. cinerea* and the oomycete *P. irregulare*. Treatments with elicitors of *P.c. carotovorum* and *Pectobacterium wasabiae* resulted in a significant increase of *PpPR-10* expression compared to control plants at 4 hours (**Figure 3A**). Inoculation with *B. cinerea* induced *PpPR-10* gene expression with maximum transcript accumulation at 24 h (**Figure 3A**), which correlates with an increase of fungal biomass (Ponce de León et al., 2012). Digital expression profiles also showed that two *PpPR-10-like* genes, Phypa_109415 and Phypa_62196, are significantly induced upon *B. cinerea* inoculation (**Supplementary Figure S3**). In contrast, no increase in *PpPR-10* transcript levels was observed when plants were inoculated with *P. irregulare*. When hormones involved in defense against biotic stress were applied on moss colonies, *PpPR-10* transcript levels increased significantly after 4 h with SA treatment, while no clear increase could be observed after treatments with MeJA, ABA, or auxin (NAA) (**Figure 3B**). Taken together, these results indicate that *PpPR-10* and *PpPR-10-like* genes are differentially regulated during moss development and pathogen infection.

### Overexpression of PpPR-10 Enhances Resistance against *Pythium irregulare* in *Physcomitrella patens*

**FIGURE 4:**
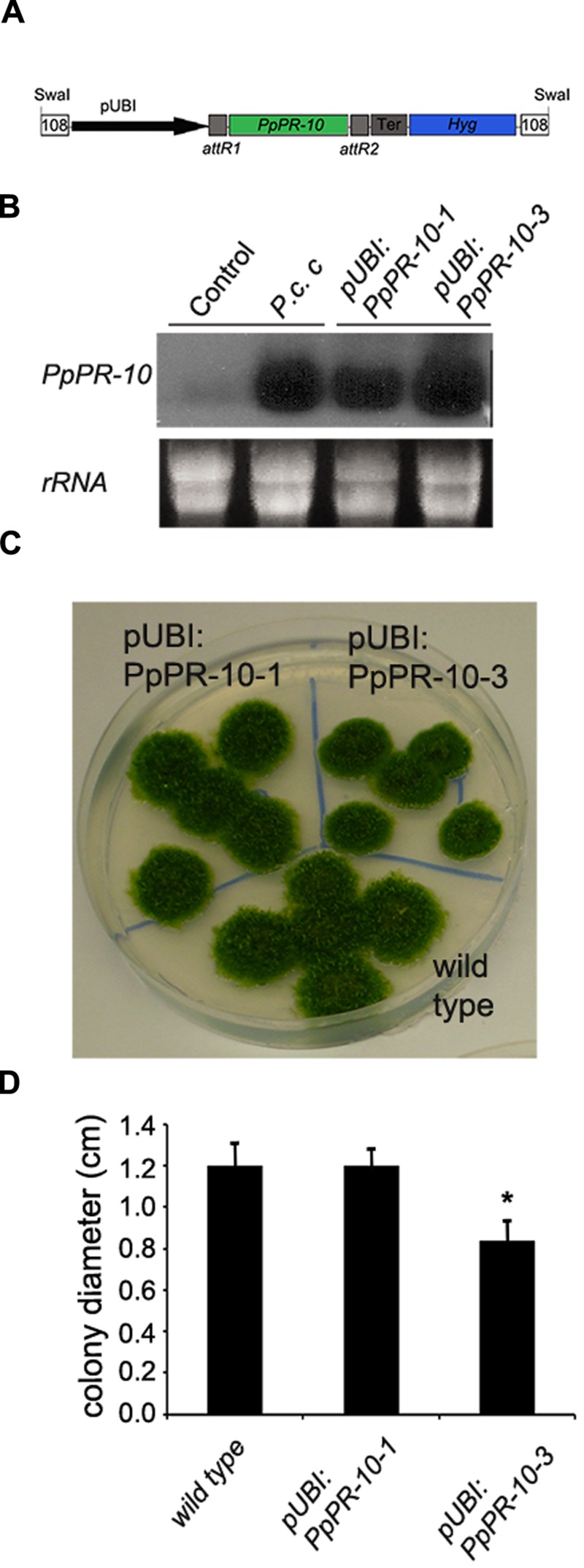
**Generation of PpPR-10 overexpression *P. patens* plants. (A)** Schematic representation of PpPR-10 overexpressing construct using plasmid pTHUbi. **(B)** Transcript levels of *PpPR-10* in untreated (Control) and *Pectobacterium corotovorum* subsp. *carotovorum* (*P.c. c*) elicitor-treated wild type plants, and untreated pUBI:PpPR-10 overexpressing lines. **(C)** Phenotype of wild type, pUBI:PpPR-10-1, and pUBI:PpPR-10-3 moss colonies. **(D)** Size of wild-type, pUBI:PpPR-10-1, and pUBI:PpPR-10-3 moss colonies grown for 21 days in BCDAT medium measured as diameter in centimeters. Results and standard deviation correspond to 16 colonies per sample. Asterisk for pUBI:PpPR-10-3 colonies indicates that the values are significantly different from wild type plants according to Kruskal–Wallis test: *P* < 0.001.

**FIGURE 5:**
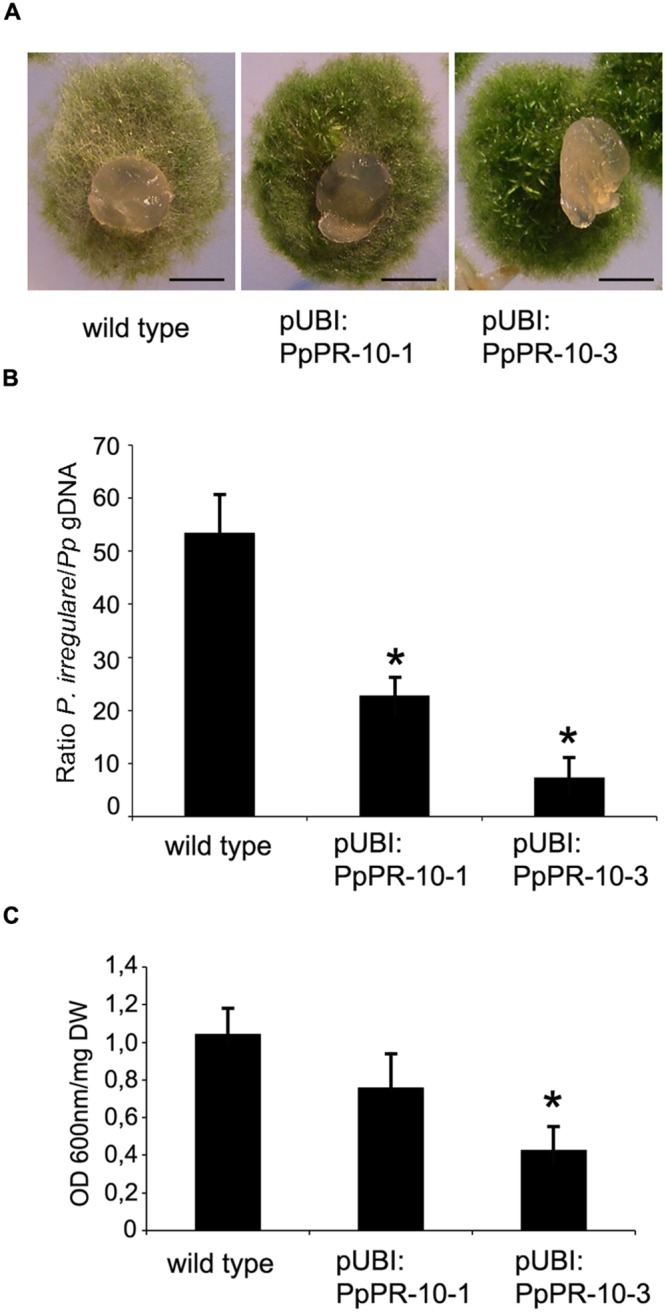
**Symptom development and mycelium growth of *P. irregulare* in wild type and PpPR-10 overexpressing moss lines. (A)** Symptom development in wild type, pUBI:PpPR-10-1 and pUBI:PpPR-10-3 moss colonies. **(B)** *P. irregulare* DNA levels were estimated by qPCR analysis. Ratios of *P. irregulare* to *P. patens* (*Pp*) gDNA were determined by qPCR with primers ITSf/ITSr and EFf/EFr, respectively. The results and standard deviation of three independent triplicate experiments are shown. **(C)** Measurement of cell death by Evans blue staining 24 h after inoculation of wild type, pUBI:PpPR-10-1 and pUBI:PpPR-10-3 moss colonies with *P. irregulare*. Data were expressed as the optical density (OD) at 600 nm per milligram of dry weight (DW). Values are means with standard deviations of six independent replicate moss samples. Experiments were repeated trice with similar results. Asterisks indicate a statistically significant difference between the wild type and overexpressing PpPR-10 plants [Students *t*-test, *P* < 0.005 (^∗^)]. The *scale bars* represent 0.5 cm.

To assess the functional role of PpPR-10 in moss defense against pathogens, we generated *P. patens* lines overexpressing PpPR-10. The cDNA sequence corresponding to *PpPR-10* was placed under the control of the maize ubiquitin promoter (Perroud et al., 2011) (**Figure 4A**), and transformed into *P. patens* protoplasts. Two transformants designated pUBI:PpPR-10-1 and pUBI:PpPR-10-3, exhibiting high constitutive level of PpPR-10 were selected for further studies (**Figure 4B**). Untreated overexpressing lines reached similar transcript levels of *PpPR-10* as wild type plants treated with elicitors of *P.c. carotovorum*, being pUBI:PpPR-10-3 the line with the highest expression level (**Figure 4B**). Haploidy of both lines was confirmed by measuring nuclear DNA content (data not shown). The phenotype of pUBI:PpPR-10-1 during gametophytic growth under normal growth conditions did not differ from the wild type, while pUBI:PpPR-10-3 colonies were somewhat smaller in size (**Figures 4C,D**). In order to analyze the possible role of PpPR-10 during defense against pathogen infection, wild type, pUBI:PpPR-10-1 and pUBI:PpPR-10-3 colonies were inoculated with *B. cinerea, P. irregulare*, or treated with elicitor of *P.c. carotovorum*, and symptom development was analyzed. *B. cinerea* inoculation and elicitor treatment did not result in differences in symptom development between the different genotypes (data not shown). However, pUBI:PpPR-10-1 and pUBI:PpPR-10-3, had less symptoms after *P. irregulare* infection, evidenced by the presence of more healthy green tissues, and apparently less mycelium growing on top of the colonies compared to wild type plants (**Figure 5A**). The observed reduction in symptom development and mycelium growth was apparently more pronounced in pUBI:PpPR-10-3 (**Figure 5A**). To confirm these results, quantitative PCR analysis was performed to quantify mycelium growth and cell death caused by *P. irregulare* infection was measured. For qPCR analysis, primers were designed to specifically amplify *P. irregulare* DNA from total DNA samples obtained from the infected plants. The results showed that after 24 h, *P. irregulare* colonization and mycelium growth was significantly higher in wild type plants compared to pUBI:PpPR-10-1 and pUBI:PR-10-3 (**Figure 5B**). In addition, cell death decreased significantly in pUBI:PpPR-10-3 compared to wild type and pUBI:PpPR-10-1, which is indicative of lower cellular damage (**Figure 5C**). No difference in cell death levels was detected when wild type or PpPR-10-1 and PpPR-10-3 were inoculated with *B. cinerea* or treated with elicitors of *P.c. carotovorum* (data not shown). Taken together, the results indicated that overexpression of PpPR-10 increased resistance against the oomycete *P. irregulare*, which was evidenced by lower mycelium growth and in case of line pUBI:PpPR-10-3 also less cellular damage, compared to wild type plants.

### Overexpression of PpPR-10 Changes Cell Wall Composition in *P. patens* Tissues

**FIGURE 6:**
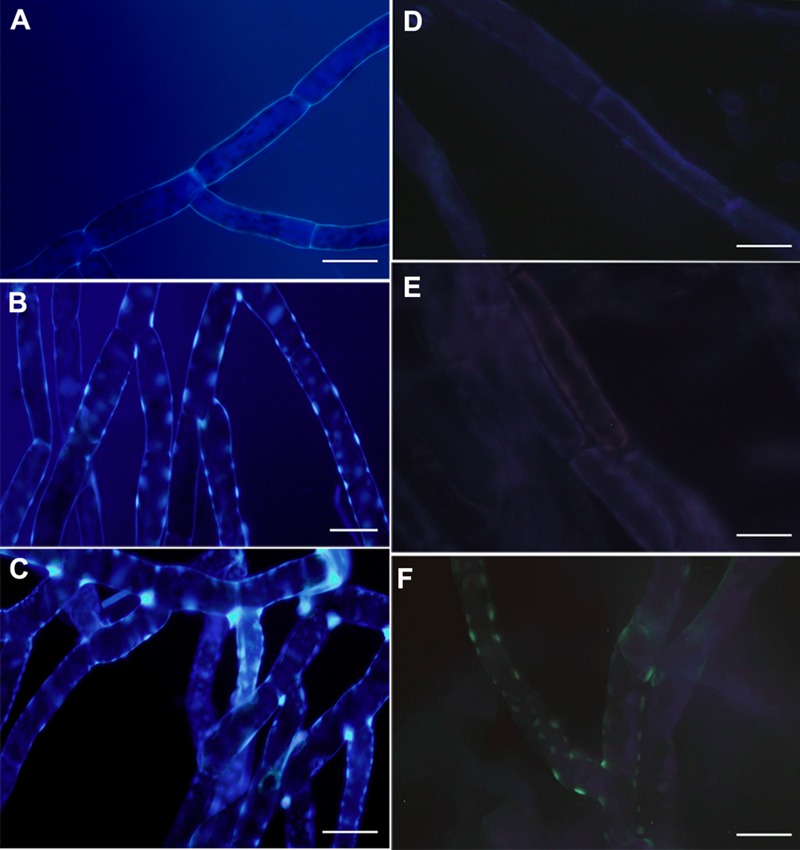
**Cell wall associated modification in overexpressing PpPR-10 *P. patens* protonemal tissues.** Untreated protonemal tissues of wild type **(A)**, pUBI:PpPR-10-1 **(B)**, and pUBI:PR-10-3 **(C)** stained with solophenyl flavine. Untreated protonemal tissues stained with methyl blue showing callose deposition in wild type **(D)**, pUBI:PpPR-10-1 **(E)**, and pUBI:PR-10-3 **(F)**. The *scale bars* represent 20 μm.

To further analyze the PpPR-10-associated defense mechanisms resulting in increased disease resistance to *P. irregulare*, we focused on cell wall modifications, since we have previously shown that *P. patens* responds to this oomycete by reinforcing the cell walls (Oliver et al., 2009). Overexpressing PpPR-10 in *P. patens* led to constitutive cell wall modification in protonemal filaments as evidenced by bright white spots in cell walls of pUBI:PpPR-10-1 and pUBI:PR-10-3 stained with solophenyl flavine (**Figure 6**). These cell wall depositions were not visualized in wild type protonemal tissues (**Figure 6**), or in leaves of the three different genotypes (data not shown). The dye solophenyl flavine binds to polysaccharides such as xyloglucan (Anderson et al., 2010). The positive staining of protonemal filaments of overexpressing plants with this dye reflects changes occurring in the cell walls. When callose deposition was evaluated using methyl blue dye in protonemal tissues of the three genotypes, only pUBI:PR-10-3 showed deposition of callose visualized as green yellow spots (**Figure 6**). Since the accumulation of ROS, especially H_2_O_2_, is associated to modification of cell walls by protein cross-linking and incorporation of phenolic compounds (Asselbergh et al., 2007), we evaluated reactive oxygen species (ROS) accumulation after *P. irregulare* infection. In wild type and PpPR-10 overexpressing lines H_2_O_2_ and superoxide accumulated after *P. irregulare* infection, although no clear differences could be observed between the different genotypes (**Supplementary Figure S4**). Taken together, these results indicate that PpPR-10 overexpression changes cell wall composition in protonemal tissues of *P. patens*, which could contribute to the increased resistance observed against *P. irregulare* in the overexpressing lines compared to wild type plants.

### Heterologous Expression of PpPR-10 in *Arabidopsis* Enhances Resistance against *P. irregulare*

**FIGURE 7:**
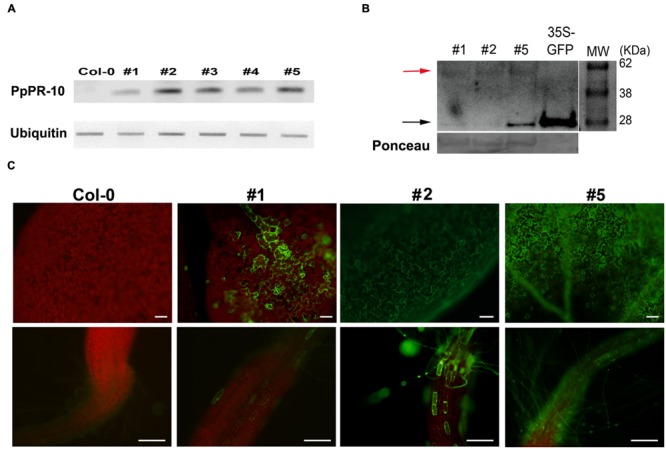
**Overexpression of PpPR-10 in *Arabidopsis*. (A)** Representative semiquantitative RT-PCR analysis of *PpPR-10* transcript levels in *Arabidopsis* PpPR-10 transformed lines. The ubiquitin gene AT3G52590 was used as an internal control. **(B)** Immunoblot detection of PpPR-10-GFP fusion protein (red arrow) in transgenic *Arabidopsis* lines At 35S:PpPR-10-1 (#1), At 35S:PR-10-2 (#2) and At 35S:PR-10-5 (#5). Ten micrograms of total soluble proteins were separated in SDS-PAGE and Western blot analysis was performed using an antibody for GFP. As a control for GFP detection, a protein sample from transgenic *Arabidopsis* plants expressing constitutively unfused GFP (35S-GFP) was included. The 27 kDa protein corresponds to cleaved GFP (black arrow). **(C)** Fluorescent microscopy of leaves and roots of wild type (Col-0), At 35S:PpPR-10-1 (#1), At 35S:PR-10-2 (#2) and At 35S:PR-10-5 (#5) *Arabidopsis* lines. The *scale bars* represent 50 μm.

**FIGURE 8:**
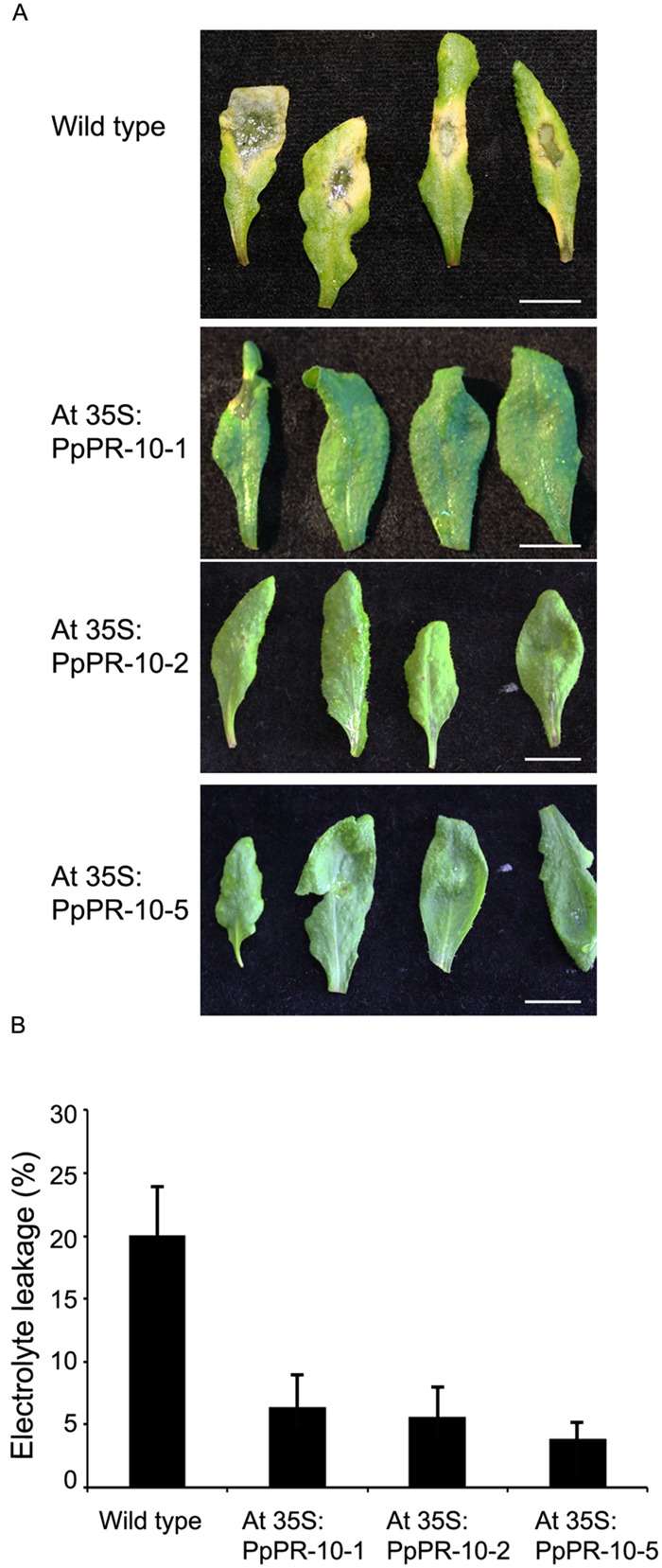
**Symptom development and ion leakage measurements in wild type and *Arabidopsis* PpPR-10 overexpressing lines. (A)** Symptom development in wild type, At 35S:PR-10-1, At 35S:PR-10-2, and At 35S:PR-10-5 leaves. **(B)** Electrolyte leakage in wild type and overexpressing PpPR-10 *Arabidopsis* plants.

**FIGURE 9:**
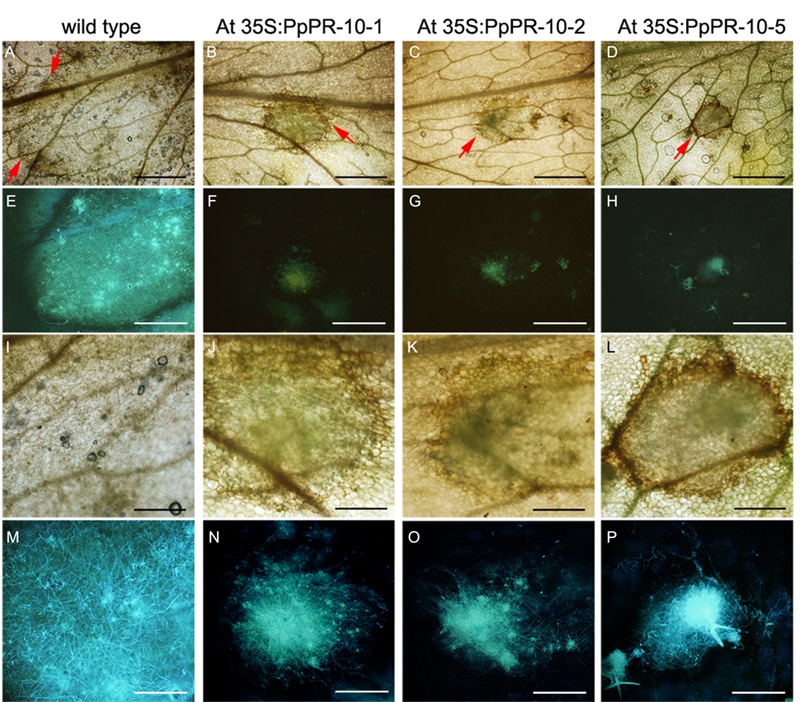
***Pythium irregulare* infected *Arabidopsis* wild type and overexpressing PpPR-10 lines.** Symptom development in 2 days-inoculated *Arabidopsis* leaves of wild type **(A)**, At 35S:PpPR-10-1 **(B)**, At 35S:PR-10-2 **(C)**, and At 35S:PR-10-5 **(D)**. Red arrows indicate the border of the lesions. Hyphae in the infected tissues were visualized using the fluorescent dye solophenyl flavine in wild type **(E)**, At 35S:PpPR-10-1 **(F)**, At 35S:PR-10-2 **(G)**, and At 35S:PR-10-5 **(H)**. Magnification of the same pictures as in **(A–G**) are shown in **(I–P)**, respectively. *Scale bars* represent in **(A–H)**; 1 mm and in **(I–P)**; 0,3 mm.

The increased resistance to *P. irregulare* observed in *P. patens* overexpressing PpPR-10 plants prompted us to evaluate the effect of the heterologous expression of this gene in the flowering plant *A. thaliana*. Stable transformed *Arabidopsis* plants overexpressing constitutively the PpPR-10-GFP fusion were generated. The presence of the transgene in the transformed plants was verified by semi-quantitative PCR of genomic DNA from 10 lines. Three lines, At 35S:PpPR-10-1, At 35S:PR-10-2, and At 35S:PR-10-5, were selected for monitoring protein fusion accumulation by Western blot using antibodies anti-GFP (**Figure 7**). GFP antibodies detected a specific band of 58 kDa in the transgenic plants, consistent with the expected size of the PpPR-10-GFP fusion protein. The additional band represented by a 27 kDa protein corresponded to cleaved GFP. Tissues of the three overexpressing PpPR-10 lines were further characterized by microscopy. All three lines showed different levels of GFP fluorescence and these levels were consistent with the semi-quantitative PCR analysis. No visible differences were observed among the wild type and the PpPR-10 transgenic *Arabidopsis* plants (data not shown). Wild type and overexpressing PpPR-10 *Arabidopsis* plants were grown in soil and after 3 weeks detached leaves were inoculated with *P. irregulare*. Two days after inoculation, symptoms were evaluated and cellular damage caused by *P. irregulare* infection was measured by ion leakage. All overexpressing PpPR-10 *Arabidopsis* lines developed less symptoms evidenced by smaller disease lesions (**Figure 8**). When the necrotic lesions were analyzed in more detail, mycelium growth was constrained in all genotypes to the lesions and pathogen proliferation associated to lesion development was higher in wild type tissues (**Figure 9**). The higher resistance of the overexpressing PpPR-10 lines compared to wild type plants was also supported by less electrolyte leakage from infected plant tissue, reflecting lower levels of cellular damage in the transgenic lines (**Figure 8**). Taken together, these results indicate that heterologous expression of PpPR-10 in *Arabidopsis* plants increases resistance against the oomycete *P. irregulare*.

## Discussion

PR-10 proteins have been shown to participate in developmental processes of flowering plants and defense responses against different types of pathogens, including virus, bacteria, and fungus (Liu and Ekramoddoullah, 2006). Here, we show that a *PR-10* gene from the moss *P. patens, PpPR-10*, is induced in response to the necrotrophic pathogens *B. cinerea* and *P.c. carotovorum*. PpPR-10 belongs to a multigene family composed of six members containing two Bet V1 domains in their predicted protein sequence, and three other members encoding proteins with a single Bet VI domain. PR-10 proteins containing two Bet VI domains have also been found in some flowering plants (Radauer et al., 2008), but the functional implication of this sequence feature is at present unknown. Comparison of the deduced protein sequences of *PpPR-10* and *PpPR-10*-like genes shows that the PR-10 conserved glycine rich GXGXXG motif is present in PpPR-10 and all moss PpPR-10-like proteins. The conserved lysine residue located 18 amino acid residues downstream the glycine-rich motif was only found in the first Bet v1 domain of the two domains containing PpPR-10 and PpPR-10-like proteins and only in the single Bet VI domain containing protein encoded by Phypa_163947. The functional implication of this result is at present unknown. Three-dimensional structure predictions revealed that each Bet v1 domains of PpPR-10 has three α helices and seven β strands, which could form a large internal hydrophobic cavity involved in ligand binding. This is in accordance with the crystal structure determined for flowering plant PR10 proteins (Fernandes et al., 2013). Phylogenetic analysis shows that moss PR-10 belongs to a basal plant cluster along with two *S. moellendorffii* homologs, while PR-10 from flowering plants and one *S. moellendorffii* homolog form a separate clade. This suggests a tendency of the moss PR-10 family to jointly evolve, showing lower intraspecific than interspecific variability among the PR-10 members, as it has been observed in *Passiflora* (Finkler et al., 2005) and in *Betula* species (Schenk et al., 2009).

Like members of PR-10 multigene families of flowering plants (Lebel et al., 2010), moss *PR-10* genes are regulated during different developmental stages, suggesting tissue specificity. A proper regulation of PR-10 gene expression in different tissues is probably needed since the *P. patens* overexpressing line exhibiting the highest *PpPR-10* transcript levels, pUBI:PpPR-10-3, was not able to develop sporophytes (data not shown). In silico analysis of the expression profile of the different moss *PR-10* genes suggested that two other members of this family are also differentially regulated by *B. cinerea*. Interestingly, these genes, *PpPR-10*, and two *Pp-PR-10-like* genes (Phypa_109415 and Phypa_62196), are grouped together in the phylogenetic tree. These results, together with a differential regulation of *P. patens PR-10* genes during abiotic stress (data not shown), suggest a functional diversification of moss *PR-10* genes during evolution, which is consistent with what has been observed in flowering plants (Koistinen et al., 2005; Handschuh et al., 2007). Interestingly, *PpPR-10* expression was induced by the defense hormone SA, but not by ABA, auxin, and MeJA. In addition, previous studies have shown that *PpPR-10* was not induced by cytokinin (Brun et al., 2004). SA regulation of this type of genes has been reported for members of the PR-10 gene family of rice and soybean (McGee et al., 2001; Xu et al., 2014). The induction of *PpPR-10* by SA is interesting since only few studies link SA to moss defense against pathogens (Andersson et al., 2005; Ponce de León et al., 2012).

Since no functional role has been assigned to *PpPR-10* and moss knockout mutants did not reveal any altered phenotype (Brun et al., 2004), we opted for an overexpression approach. Only the line exhibiting the highest expression level of PpPR-10 showed an altered growth rate during gametophytic development. Both PpPR-10 overexpressing moss lines showed similar symptom development and cellular damage to *B. cinerea* and *P.c. carotovorum* elicitor treatments compared to wild type plants (data not shown). Despite the fact that *P. irregulare* did not induce *PpPR-10* expression in wild type plants, overexpression of this gene enhanced resistance to this necrotrophic oomycete in *P. patens*. Moreover, heterologous expression of *PpPR-10* in *Arabidopsis* plants also improved resistance to *P. irregulare* infection. Overexpression of *PR-10* genes in flowering plants has resulted in different phenotypes. While transgenic *Gossypium hirsutum* PR-10 expressing *Arabidopsis* lines and pea PR10.1 expressing *Brassica napus* plants did not show improved resistance to pathogens (Wang et al., 1999; Chen and Dai, 2010), overexpressing pea PR-10.1 in potato, and soybean GmPRP in tobacco and soybean conferred resistance to *Verticillium dahliae* and *Phytophthora*, respectively (Chang et al., 1993; Jiang et al., 2015). The precise function of PR-10 proteins remains unknown, however, some PR-10 proteins exhibit antimicrobial activity, DNase and/or RNase activity or bind cytokinin or other ligands, including fatty acids and flavonoids (Fernandes et al., 2013; He et al., 2013). PpPR-10 from the moss *P. patens* was previously reported as UBP34 (urea-type cytokinin-binding protein of 34 kDa) (Gonneau et al., 2001) and a second PpPR-10-like protein (Phypa_109415) has been shown to bind cytokinin in this moss (Brun et al., 2004). The relevance of the cytokinin binding capacity of PpPR-10, PpPR-10-like and other PR-10 proteins in plant defense against pathogens is currently unknown. Interestingly, overexpression of a pea *PR-10* gene in *Arabidopsis* plants leads to higher cytokinin content and increased expression of genes upregulated by cytokinin, providing additional evidence for a role for PR-10 in cytokinin homeostasis, which in turn may modulate PR-10 function (Srivastava et al., 2007; Krishnaswamy et al., 2008). However, pUBI:PpPR-10 overexpressing *P. patens* plants did not show any phenotype associated with an altered cytokinin content, such as an increase in the number of buds in protonemal tissue (Reski and Abel, 1985). Cytokinins regulate plant growth and development and have been shown to participate in plant defense against pathogens (Choi et al., 2010; Naseem et al., 2015). Induction of cytokinin-mediated responses has been shown to enhance plant defense against necrotrophic pathogens (Swartzberg et al., 2008; Choi et al., 2010). On the other hand, cytokinin binding capacities of some PR-10 have been associated to RNase activity (Krishnaswamy et al., 2008). Zubini et al. (2009) have shown that RNA hydrolyzing activity of a PR-10 from peach could regulate the endogenous cytokinin concentration and that this ligand could have a possible regulatory role on the PR-10 enzymatic activity through a feedback mechanism. Further studies are needed to reveal if PpPR-10 has RNase activity and if this is associated to cytokinin binding, modulating PpPR-10 functions.

*Pythium irregulare* and other *Pythium* species directly penetrate their host (Oliver et al., 2009), and therefore cell wall composition is probably an important factor for plant resistance against these types of pathogens. Consistently, cell wall strengthening was differentially observed in rice roots infected with *Pythium* species with different virulence (Van Buyten and Höfte, 2013). *P. irregulare* infection elicits cell wall modifications in *P. patens* cells that are stained with solophenyl flavine and safranine-O staining (Oliver et al., 2009), which detect xyloglucan, and incorporation of phenolic compounds, respectively. The bright white spots identified in solophenyl flavine stained cell walls of untreated PpPR-10 overexpressing moss lines are indicative of cell wall changes, which could contribute to the reinforcement of the cell wall. Interestingly, Berry et al. (2016) have shown that xyloglucan is only present in undivided *P. patens* protoplasts with thicker cell walls. In addition, alterations in xyloglucan structure result in increased resistance against necrotrophic fungi in flowering plants (Delgado-Cerezo et al., 2012). Further studies are needed to identify a relation with the cell wall modifications detected with solophenyl flavine staining and moss resistance against this oomycete. Like in *Arabidopsis* (Adie et al., 2007), *P. patens* induces callose deposition in response to *P. irregulare* infection (Oliver et al., 2009). Adie et al. (2007) have shown that the callose-deficient mutant *pmr4* is more susceptible to this oomycete compared to wild type plants, demonstrating that callose deposition plays a role in the defense response against *P. irregulare*. Consistently, constitutive callose depositions in protonemal tissues of the overexpressing line pUBI:PR-10-3 could contribute to the higher resistance observed against this oomycete. In contrast, we did not observe cell walls changes in leaves of PpPR-10 overexpressing *Arabidopsis* plants stained with safranine-O or solophenyl flavine, suggesting that the increased resistance to *P. irregulare* observed in *Arabidopsis* is not related to cell wall modifications detected by these dyes. The accumulation of ROS, especially H_2_O_2_, is associated to cell wall modifications by incorporation of phenolic compounds and protein cross-linking (Asselbergh et al., 2007), In PpPR-10 overexpressing moss plants, ROS accumulation could not be related to the cell wall modifications observed (**Supplementary Figure S4**), and in *Arabidopsis* H_2_O_2_ and superoxide accumulation increase after *P. irregulare* infection, although no differences could be detected between the wild type and the overexpressing PpPR-10 plants (data not shown). According to the digital expression profiles available in the Phytozome database, *PpPR-10* transcript accumulation correlates with the expression of genes coding for enzymes of the phenylpropanoid pathway, such as phenylalanine ammonia-lyase and enzymes involved in cell wall synthesis, like cellulose synthase and pectinesterase (data not shown). Temporally and spatially correlation between expression of *PR-10* genes and other genes related to the phenylpropanoid pathway from flowering plants, has been previously reported. This raises the possibility that PR-10 proteins act in concert with the phenylpropanoid pathway, or that phenylpropanoid intermediates induce PR-10 genes (Zubini et al., 2009). Muñoz et al. (2010) suggest that members of the strawberry PR-10 protein family play an important role in the control of phenylpropanoids and flavonoids biosynthesis. Strawberry PR-10 binds flavonoids and therefore this protein could act as a “chemical chaperone” binding to flavonoid intermediates and making them available to processing enzymes (Casañal et al., 2013). Birch PR-10 also binds flavonoids, including flavone, and flavanone (Mogensen et al., 2002), and flavonoid glycosides, which can be located at the cell wall (Koistinen et al., 2005). These authors suggest that birch PR-10 could be involved in the storage of flavonoids and their transport to the site where they are needed (Koistinen et al., 2005). In addition, overexpression of a *PR-10* gene from *G. hirsutum* in *Arabidopsis* causes an increase in total flavonoid content (Chen and Dai, 2010). Thus, it will be relevant to evaluate the role of PpPR-10 in flavonoid and/or phenolic compounds content, binding, and/or transportation. Elucidation of the chemical composition of the bright white spots detected in protonemal filaments of the overexpressing lines will help to identify possible changes occurring in metabolic pathways in *P. patens*.

Previous studies have shown that in response to *P. irregulare* infection *Arabidopsis* activates defenses predominantly mediated by JA and ABA, while SA is required but to a lesser extent (Adie et al., 2007). In this plant JA, SA, and ABA levels increase after *P. irregulare* infection (Adie et al., 2007). In contrast, in *P. irregulare-*infected *P. patens* tissues SA does not increase and the small amount of JA detected is probably synthesized by the pathogen since JA is not produced in this moss (Oliver et al., 2009; Ponce de León et al., 2012). A group of *Arabidopsis* genes was identified as being dependent on both *P. irregulare* infection and at least one of the JA, ABA, and SA hormonal defense pathways (Adie et al., 2007). When the expression levels of some of these genes were analyzed in uninfected and infected PpPR-10 overexpressing *Arabidopsis* plants, including PDF1.2a (JA-dependent), PR-1 (SA-dependent), and tropinone reductase and protein phosphatase 2C (ABA-dependent), no clear changes could be observed between the different genotypes, suggesting that the increased resistant observed in the overexpressing plants is not due to alterations of these pathways and/or enhanced expression of these genes (data not shown). In summary, the results presented here indicate that PpPR-10 confers resistance in *P. patens* and in *Arabidopsis* against the oomycete *P. irregula*re. Further characterization of PpPR-10 function under biotic stress will enhance our understanding of the molecular mechanisms mediating plant defense responses.

## Author Contributions

AC conducted most experiments. SV participated in the PpPR-10 subcellular localization analysis and helped to draft the manuscript. IPDL designed and supervised the study, performed the inoculation of the *Arabidopsis* plants and the corresponding histological analysis, contributed to the analysis of the data and wrote the manuscript. All authors read and approved the final manuscript.

## Conflict of Interest Statement

The authors declare that the research was conducted in the absence of any commercial or financial relationships that could be construed as a potential conflict of interest.

## Footnotes

1 http://zhanglab.ccmb.med.umich.edu/I-TASSER/

2 http://gimp.org

3 https://www.genevestigator.com

## Supplementary Material

FIGURE S1 **Three-dimensional model of PpPR-10.** The model was built using the I-TASSER server. From five PpPR-10 models predicted, the best model is shown with a confidence score (*C*-score) of 0.49.

FIGURE S2 **Phylogenetic analysis of deduced PR-10 proteins in *P. patens* and other plants.** Full-length amino acid sequences were aligned using ClustalW algorithm and a phylogenetic tree was constructed by the neighbor-joining method using MEGA version 5.05. Numbers at branch nodes represent the confidence level of 1000 bootstrap replications. The identities of the protein sequences are indicated by their locus name according to Phytozome. The *P. patens* PR-10 proteins containing two Bet v1 domains are highlighted with a red spot. The abbreviations of species are as follows: Sm, *Selaginella moellendorffii*; Gm, *Glycine max*; Egr, *Eucalyptus grandis;* Stu, *Solanum tuberosum*; Aco, *Aquilegia coerulea Goldsmith*; Vvi, *Vitis vinifera*.

FIGURE S3 **Expression profiles of *P. patens PR-10* genes during development and *B. cinerea* inoculation.** Transcript levels of *P. patens PR-10* genes are presented as heat maps generated at Genevestigator based on microarray data. Values are expressed in percent of potential expression **(A)** and log-scale of potential expression **(B)** of each gene.

FIGURE S4 **Reactive oxygen species (ROS) accumulation in wild type and PpPR-10 overexpressing plants inoculated with *P. irregulare*.** DAB and NBT staining of control and *P. irregulare*-inoculated moss colonies performed after 24 h. Representative colonies are shown. *Scale bars* represent 0.5 cm.

## Abbreviations

ABA: abscisic acid
*B. cinerea*: *Botrytis cinerea*
GFP: green fluorescent protein
NAA: 1-naphthaleneacetic acid
MeJA: methyl jasmonate
*P. patens*: *Physcomitrella patens*
*P. irregulare*: *Pythium irregulare*
P.c. carotovorum: *Pectobacterium carotovorum* subsp. *Carotovorum*
PDA: potato dextrose agar
PR: pathogenesis-related protein
Prx34: peroxidase 34
SA: salicylic acid
TSPO1: tryptophan rich sensory protein
