# Supplementary figures and images for: Moss Pathogenesis-Related-10 Protein Enhances Resistance to Pythium irregulare in Physcomitrella patens and Arabidopsis thaliana
Source: Front Plant Sci. 2016 Apr 29;7:580. doi: 10.3389/fpls.2016.00580 (PMC4850436; doi:10.3389/fpls.2016.00580)

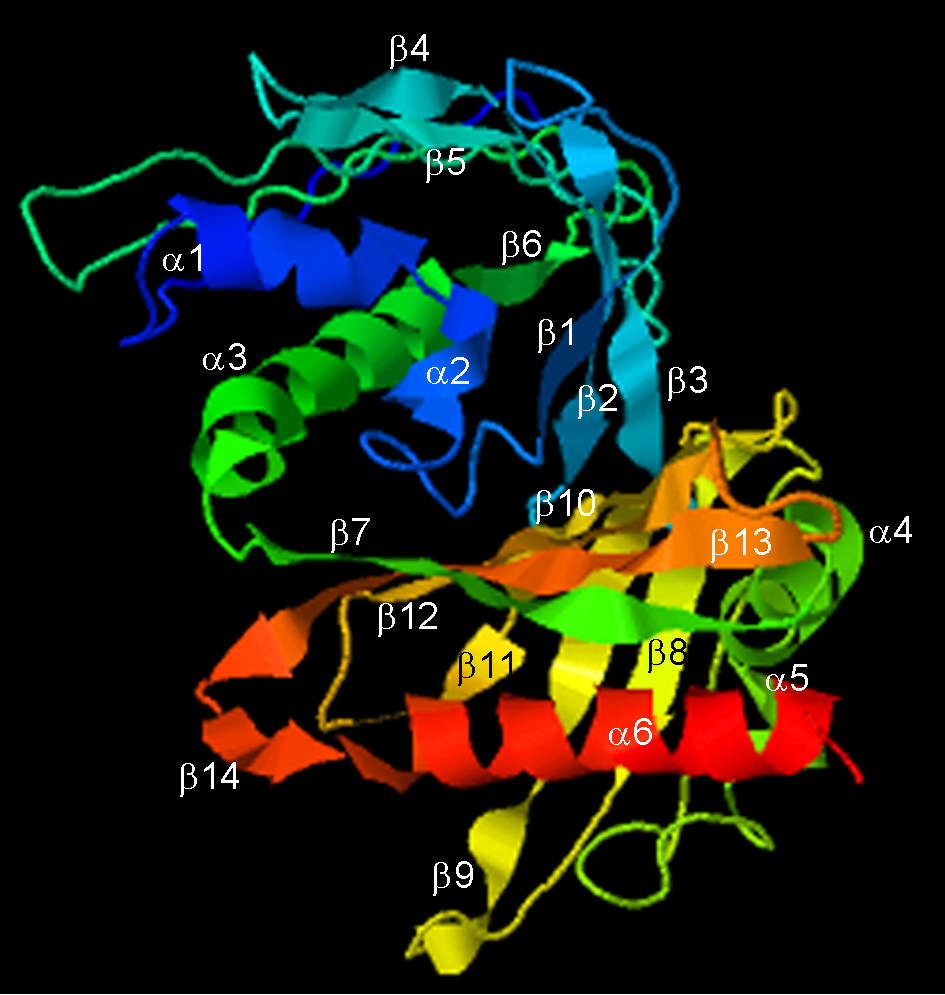

Supplement: FIGURE S1 — Three-dimensional model of PpPR-10. The model was built using the I-TASSER server. From five PpPR-10 models predicted, the best model is shown with a confidence score (C-score) of 0.49. [file Image_1.TIF]

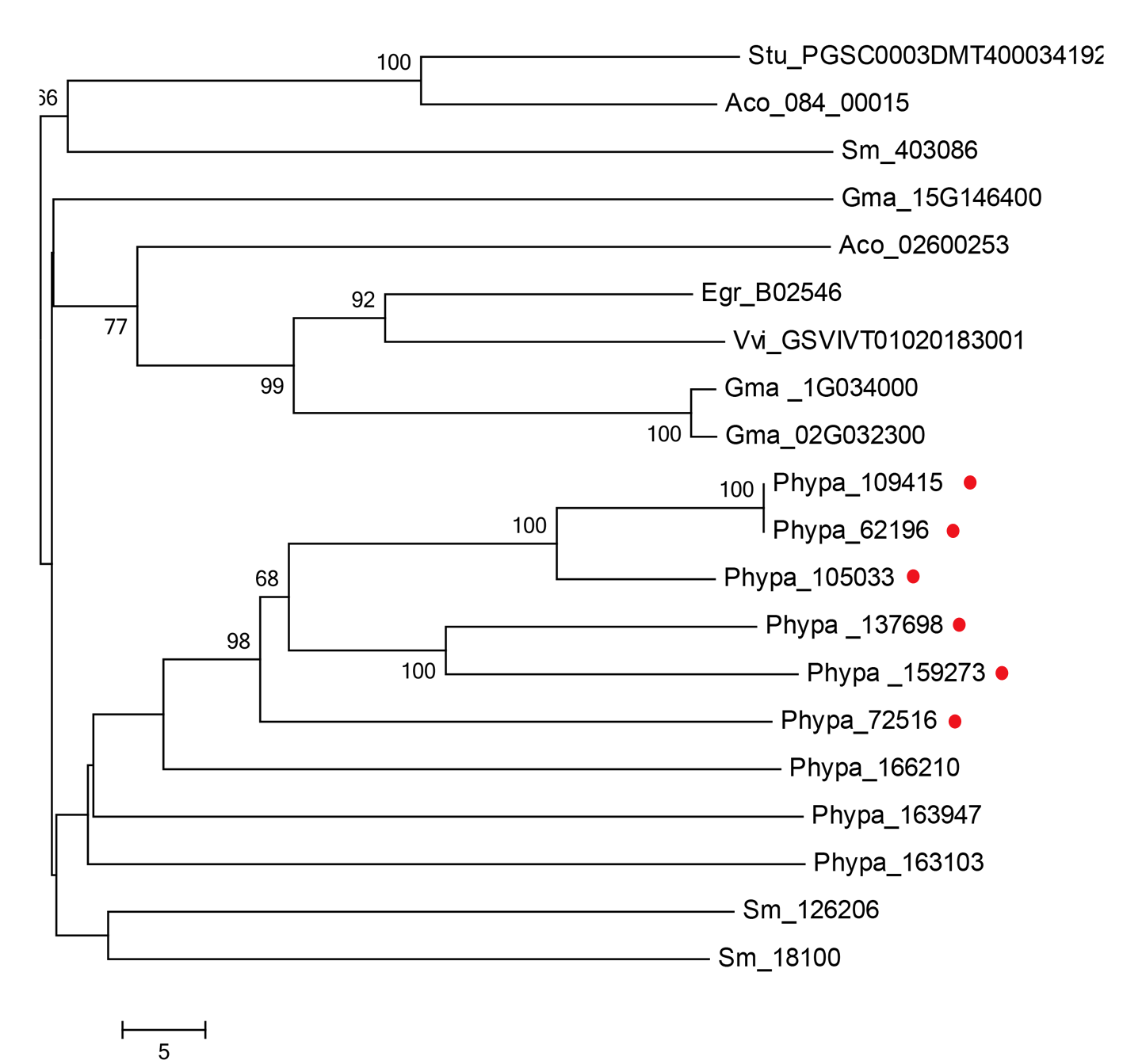

Supplement: FIGURE S2 — Phylogenetic analysis of deduced PR-10 proteins in P. patens and other plants. Full-length amino acid sequences were aligned using ClustalW algorithm and a phylogenetic tree was constructed by the neighbor-joining method using MEGA version 5.05. Numbers at branch nodes represent the confidence level of 1000 bootstrap replications. The identities of the protein sequences are indicated by their locus name according to Phytozome. The P. patens PR-10 proteins containing two Bet v1 domains are highlighted with a red spot. The abbreviations of species are as follows: Sm, Selaginella moellendorffii; Gm, Glycine max; Egr, Eucalyptus grandis; Stu, Solanum tuberosum; Aco, Aquilegia coerulea Goldsmith; Vvi, Vitis vinifera. [file Image_2.TIF]

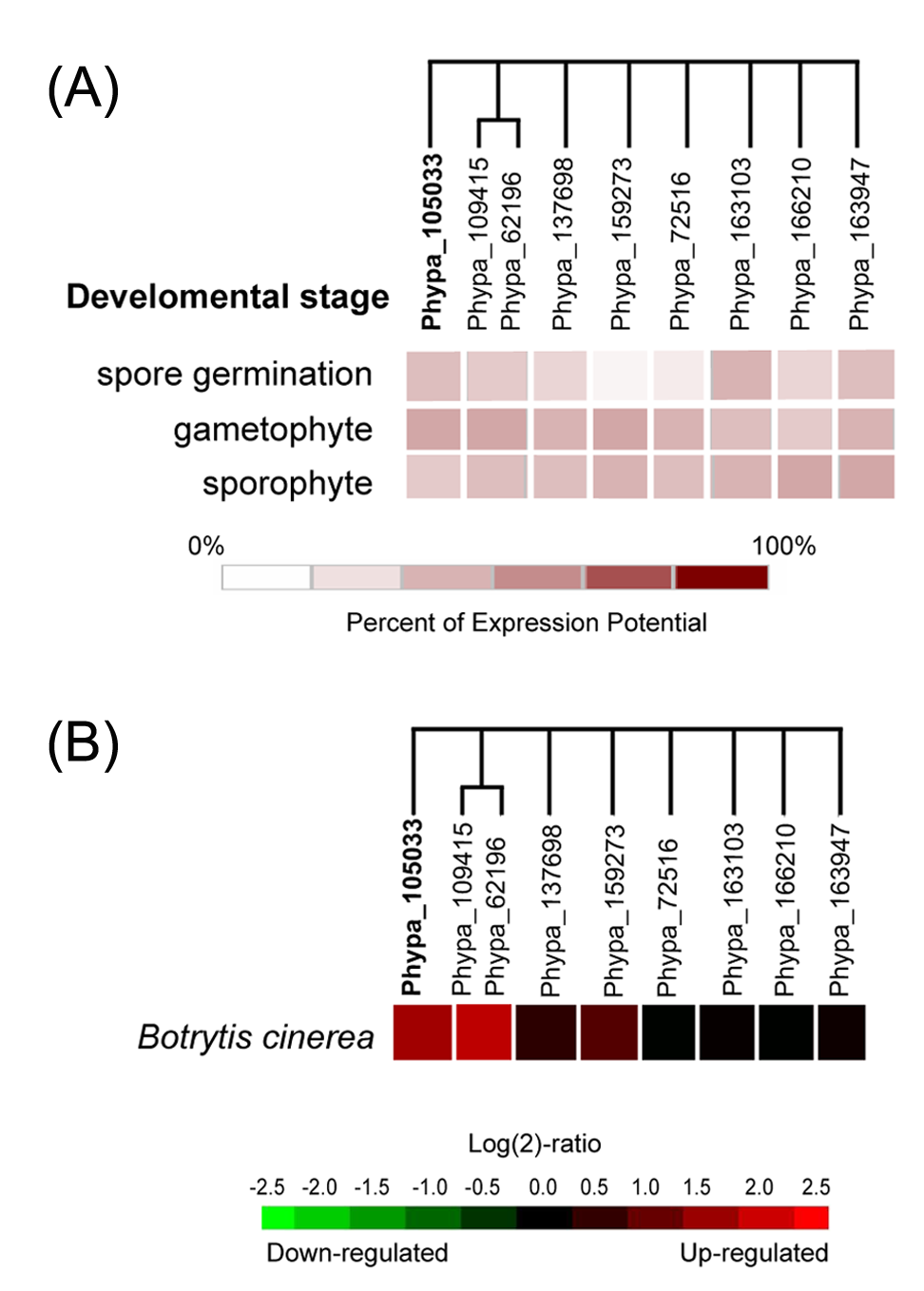

Supplement: FIGURE S3 — Expression profiles of P. patens PR-10 genes during development and B. cinerea inoculation. Transcript levels of P. patens PR-10 genes are presented as heat maps generated at Genevestigator based on microarray data. Values are expressed in percent of potential expression (A) and log-scale of potential expression (B) of each gene. [file Image_3.TIF]

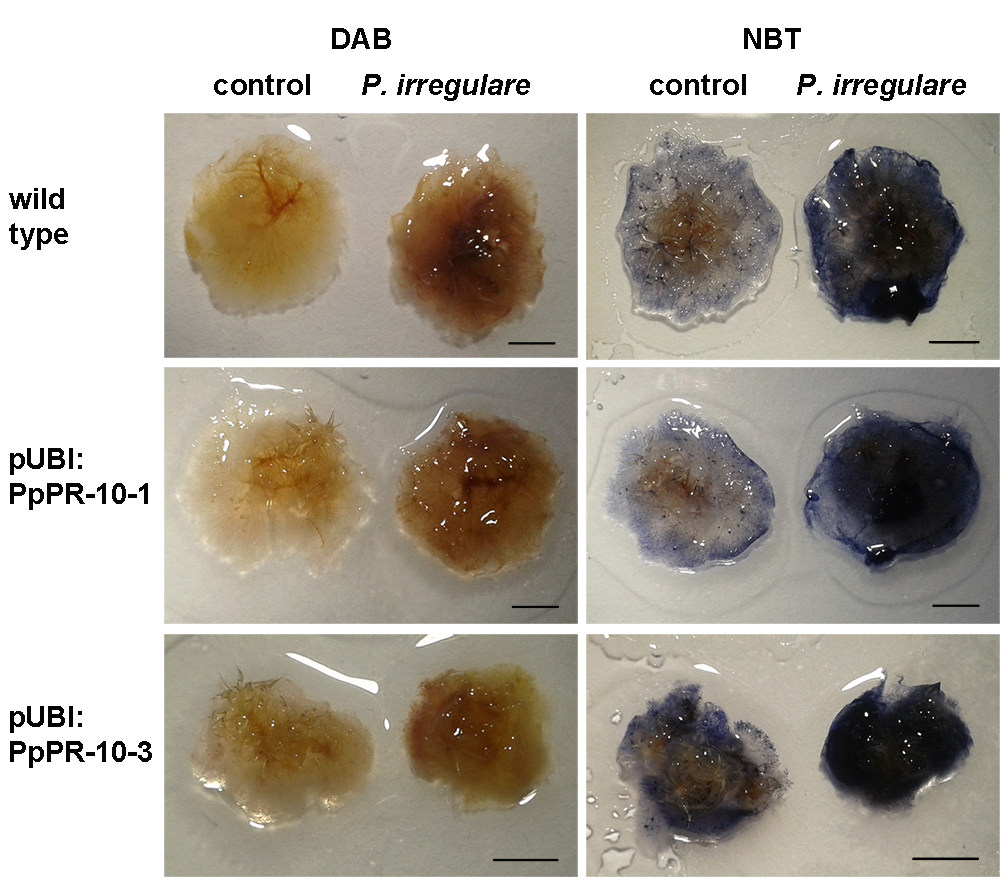

Supplement: FIGURE S4 — Reactive oxygen species (ROS) accumulation in wild type and PpPR-10 overexpressing plants inoculated with P. irregulare. DAB and NBT staining of control and P. irregulare-inoculated moss colonies performed after 24 h. Representative colonies are shown. Scale bars represent 0.5 cm. [file Image_4.TIF]
